# Exploring the Role of Empathy as a Dual Mediator in the Relationship between Human–Pet Attachment and Quality of Life: A Survey Study among Adult Dog Owners

**DOI:** 10.3390/ani13132220

**Published:** 2023-07-06

**Authors:** Ji Yu Sung, Jin Soo Han

**Affiliations:** 1 Department of Bio and Healing Convergence, Graduate School, Konkuk University, Seoul 05029, Republic of Korea; iacaroma@konkuk.ac.kr; 2 Department of Laboratory Animal Medicine, Institute for the 3Rs & Animal Welfare, College of Veterinary Medicine, Konkuk University, Seoul 05029, Republic of Korea

**Keywords:** empathy, pet attachments, quality of life, affective empathy, cognitive empathy, animal companionship, human–animal relationships

## Abstract

**Simple Summary:**

This study examines the relationship between human–dog attachment, empathy, and human quality of life. The findings suggest that attachment to dogs and concerns for empathy have a significant double-mediation effect on human quality of life. However, cognitive empathy does not show a significant effect. The study highlights the importance of attachment and empathy towards dogs in enhancing the quality of life of both humans and dogs and suggests a shift in perceiving dogs as independent individuals. Future research should focus on affective empathy to improve relationships and the quality of life of both humans and dogs.

**Abstract:**

This study investigates the impact of empathy on the relationship between human–dog attachment and human quality of life. A survey involving 263 dog owners was conducted to gather data on attachment to dogs, empathy, and human quality of life in Korea. The findings indicate significant correlations between attachment to dogs, human empathy, and quality of life. Specifically, both general attachment and concerns for animal rights/welfare demonstrate meaningful parallel double-mediation effects. However, cognitive empathy does not show a significant double-mediation effect on human quality of life. These findings emphasize the importance of attachment and empathy towards dogs in enhancing the quality of life of both humans and dogs. The study suggests a shift in perceiving dogs as independent individuals rather than mere substitutes for humans. Future research should focus on emotional factors, particularly affective empathy, to further enhance the quality of life for both humans and dogs through improved relationships.

## 1. Introduction

The intricate relationship between humans, attachment and animals has been the subject of extensive research, delving into various aspects including deep interactions, empathy development, and animal welfare [1,2,3,4]. The term pets has evolved into companion animals to reflect their significant role and emotional connections with humans [5,6,7]. Strong attachment to companion animals positively impacts overall quality of life, providing emotional support, enhancing psychological well-being, and promoting social relationships [8,9,10,11,12]. This bond shares similarities with human–human relationships, and pets are often considered cherished family members [13,14].

The attachment and interactions between humans and companion animals significantly impact human empathy, fostering emotional problem-solving and social connectedness [15,16,17,18]. Engaging with companion animals evokes cognitive and affective empathy, which are crucial for social relationships and psychological well-being [19,20,21,22]. The bond between children and companion animals also contributes to the development of children’s empathy [21,23,24,25]. Understanding and empathizing with the emotions of companion animals are essential for cultivating closer relationships, effective communication, and mutual understanding [26]. This empathetic bond strengthens the attachment between humans and companion animals, positively influencing our lives [27].

Spending time with companion animals has favorable impacts on both psychological and physical health, including alleviating depression and anxiety [18,27,28,29]. Companion animals provide social support, as evidenced by animal-assisted activities and pet-friendly workplaces, which enhance employee satisfaction and productivity [2,30]. Especially during the COVID-19 pandemic, companion animals served as a form of social support, preserving the identity of their owners in challenging situations [2,4,22].

Anthropomorphism and humanization play significant roles in human–animal relationships [31,32]. Anthropomorphism involves attributing human characteristics to animals, driven by emotional attachment and intimacy with companion animals. It treats animals as if they possess human-like qualities [33,34,35]. On the other hand, humanization is the projection of human thoughts, emotions, and behaviors onto animals, often due to a deep attachment. While anthropomorphism can have positive effects on the human–animal relationship, humanization can be detrimental to animal well-being due to unrealistic expectations or inappropriate treatment [4,35]. Understanding these psychological processes is crucial for the overall quality of life of both humans and companion animals [2,35].

The impact of companion animal attachment on an individual’s quality of life emphasizes the need to understand the underlying mechanisms that drive this connection [2,15,33,36]. Previous research has primarily focused on the effects of companion animal attachment. However, limited attention has been given to exploring the specific role of empathy in mediating this influence [4,26].

In Korea, dogs are the most kept pets, and this study specifically focuses on adults who own dogs. Korea has a wide range of dog breeds, from small breeds like Maltese dogs or Poodles to large breeds like Jindo dogs or Golden Retrievers [37]. Moreover, Korea’s unique pet culture emphasizes the bond between humans and their canine companions, regardless of breed or dog size, and it transcends constraints such as living space or breed characteristics. Dogs are considered family members, and this cultural aspect further supports the importance of understanding the role of empathy in the connection between humans and their dogs.

This study utilized online surveys as a method of data collection due to the challenges posed by COVID-19, which made face-to-face interviews difficult. Participants were requested to provide general demographic information and respond to questions regarding pet attachment, empathy, and quality of life. Specifically, this research examined the impact of three sub-factors of pet attachment, namely general attachment, person substitution, and animal rights/welfare, as well as two sub-factors of empathy, cognitive empathy and affective empathy, on the overall quality of life. The primary objective of this study is to shed light on the intricate and dynamic relationship between pet attachment, empathy, and quality of life, thereby contributing to the existing understanding of human–animal bonds and individual happiness. Through this research, we aim to gain further insights into the positive influence that dogs have on the lives of individuals within Korean society.

## 2. Materials and Methods

### 2.1. Questionnaires

The questionnaire had 88 questions divided into four sections: 11 demographic questions, 23 questions on the Korean-translated Lexington Attachment to Pets Scale (LAPS) [38,39,40,41], 28 questions about the Korean version of the Interpersonal Reactivity Index (IRI) [42,43], and 26 questions about the Korean version of the World Health Organization Quality of Life-Bref (WHOQOL-BREF) [44,45,46,47]. All questions used a 5-point Likert scale, with higher scores indicating more positive outcomes.

### 2.2. Data Collection

The study received approval from the Committee on Bioethics (IRB) of Konkuk University in Korea (approval number 7001355-202208-HR-577, issued on 15 September 2022), in accordance with the Declaration of Helsinki. A total of 300 participants took part in the survey, which commenced on 15 September 2022. As a gesture of gratitude, mobile coupons were provided as incentives. After excluding 37 non-responsive submissions, the final analysis included 263 respondents who owned various dog breeds, such as Maltese dogs, Pomeranians, Poodles, Mixed breed dogs, Chihuahuas, Shih Tzus, Bichon Frises, as well as other breeds including Golden Retrievers and Jindo dogs.

### 2.3. Statistical Analysis

Data collected underwent factor, reliability, descriptive, and correlation analyses using IBM SPSS/WIN ver. 29.0 (IBM Corp., Armonk, NY, USA). The PROCESS macro model No. 4 was used to test the hypotheses of the study, examining the double-mediation effect. Bootstrapping samples were set to 10,000 for repeated restoration and extraction.

### 2.4. Model

**Figure 1:**
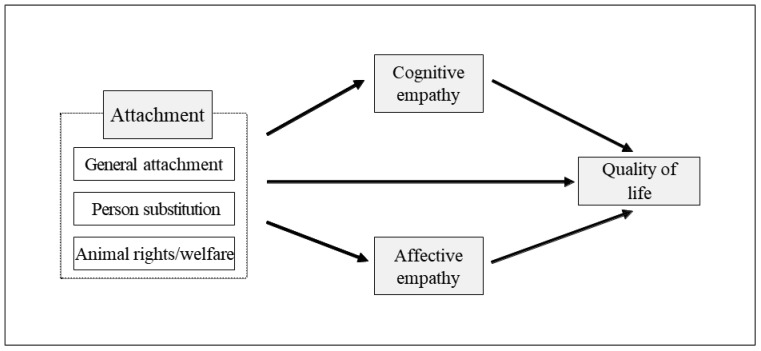
A flow diagram illustrating the effect of empathy on the relationship between human attachment to dogs and the quality of human life.

This study used a model to look at how empathy affects the relationship between attachment to dogs and human quality of life. Specifically, cognitive and affective empathy were examined as two sub-factors of empathy (see Figure 1). Three research hypotheses were presented based on this.

**H1.**  *Empathy has a dual-mediating effect on the relationship between attachment and quality of life.*

**H2.**  *Empathy has a similar effect on the relationship between person substitution for dogs and human quality of life.*

**H3.**  *Empathy has a dual effect on the relationship between animal rights/welfare and human quality of life.*

### 2.5. Validity and Reliability Verification of Measurement Results

To assess the validity and reliability of the three measurement tools utilized in this study, namely the LAPS, IRI, and WHOQOL-BREF scales, a factor analysis and a reliability analysis were conducted. However, it is important to note that for the WHOQOL-BREF scale, which was employed as the dependent variable, a factor analysis was omitted. This decision was made because, contrary to prior research, the WHOQOL-BREF scale was considered to have a single-factor structure in this study.

A principal component analysis and the Varimax rotation method were used for the factor analysis. The number of factors in each variable was determined by extracting those with an eigenvalue of 1 or higher. In the LAPS factor analysis conducted on independent variables, the model’s suitability was confirmed by a Kaiser–Meyer–Olkin (KMO) value of 0.928 and Bartlett’s sphericity test result of x2 = 3650.617 (*p* < 0.001). Three factors were identified: general attachment (eigenvalue = 5.640, accounting for 24.521% of the variance), person substitution (eigenvalue = 4.190, accounting for 18.218% of the variance), and animal rights/welfare (eigenvalue = 3.739, accounting for 16.257% of the variance). Collectively, these factors accounted for 58.996% of the variance (Table S1). The factor structure demonstrated validity, as all 23 items of the LAPS scale had factor loadings of 0.40 or higher [48].

An IRI factor analysis was conducted on the independent variables, which showed the suitability of the model with a KMO value of 0.890 and Bartlett’s sphericity test yielding x2 = 3542.758 (*p* < 0.001). Two factors were identified based on an eigenvalue of ≥1. The cognitive empathy factor accounted for 22.564% of the variance, with an eigenvalue of 6.318, while the affective empathy factor accounted for 21.222% of the variance, with an eigenvalue of 5.942. All 28 items on the IRI scale met the validity criteria for the factor structure, and together, the two factors explained 43.786% of the variance (Table S3).

Cronbach’s α coefficient was calculated to assess the reliability, with a value of ≥0.6, indicating no significant issues [49]. The general attachment factor showed a coefficient of 0.921, the person substitution factor had a coefficient of 0.841, and the animal rights/welfare factor obtained a coefficient of 0.860 (Table S2). For the cognitive empathy factor, the coefficient was 0.899, while the affective empathy factor yielded a coefficient of 0.881 (Table S3). The WHOQOL-BREF factor demonstrated a coefficient of 0.950, confirming the reliability of all variables and sub-factors examined in this study.

## 3. Results

### 3.1. Participant

Informed consent was obtained from all participants in the study, comprising 63.1% females and 36.9% males. The majority were in their 30s (58.6%) and first-time pet owners (81%). Preferred dog breeds included Poodles (20.2%), mixed breeds (20.2%), Maltese dogs (19%), Pomeranians (19%), Shih Tzus (26%), Chihuahuas (13%), Bichon Frises (11%), and other breeds (7%) such as Golden Retrievers and Jindo dogs (Table S1).

### 3.2. Results of the Descriptive Statistical Analysis of the Study Variables

Descriptive statistics were analyzed to determine the mean, standard deviation, skewness, and kurtosis of attachment to dogs, empathy, and human quality of life (Table 1). The average attachment scores were 4.35 (SD = 0.590) for overall attachment, 4.01 (SD = 0.680) for person substitution, and 4.21 (SD = 0.750) for animal rights/welfare. For empathy, the average scores were 3.55 (SD = 0.680) for cognitive empathy and 3.50 (SD = 0.660) for affective empathy. All variables met the normality assumption, with skewness values ranging from 0.013 to 1.143 and kurtosis values ranging from 0.027 to 1.038, indicating suitability for parametric statistical methods.

### 3.3. Results of Correlation Analysis between Study Variables

A correlation analysis examined the relationships between attachment to dogs, empathy, and human quality of life (Table 2). Significant positive correlations were found between attachment to dogs and human quality of life, with correlation coefficients (r) ranging from 0.472 to 0.537.

Both cognitive empathy and affective empathy showed significant positive correlations with human quality of life (r = 0.390 and r = 0.534, respectively). Significant positive correlations were also observed between attachment to dogs and empathy sub-factors, with cognitive empathy ranging from 0.275 to 0.454 and affective empathy ranging from 0.368 to 0.424.

### 3.4. The Parallel Dual-Mediating Effect of Empathy on the Relationship between General Attachment and Human Quality of Life

The analysis confirmed Hypothesis 1 by examining the dual-mediation effect of empathy sub-factors on general attachment and human quality of life. Regression models were found to be suitable based on F-statistics (mediation model 1: F = 24.494, mediation model 2: F = 45.319, dependent variable model: F = 63.154) (Table 3).

In the dependent variable model, general attachment had positive effects on human quality of life (t = 6.818, *p* < 0.001), cognitive empathy (t = 2.753, *p* < 0.01), and affective empathy (t = 6.181, *p* < 0.001). A mediation analysis revealed significant positive effects of general attachment on cognitive empathy (t = 4.949, *p* < 0.001) and affective empathy (t = 6.732, *p* < 0.001), explaining 8.6% and 14.8% of the variance, respectively.

Furthermore, attachment to animals positively influenced human quality of life, cognitive empathy, and affective empathy, explaining 42.2% of the variance (t = 6.818, *p* < 0.001; t = 2.753, *p* < 0.01; t = 6.181, *p* < 0.001). Bootstrap estimates confirmed these relationships. Animal rights/welfare significantly influenced cognitive empathy (t = 4.613, *p* < 0.001) and affective empathy (t = 7.554, *p* < 0.001), mediating the relationship between animal rights/welfare and human quality of life. The animal rights/welfare variable showed significant positive associations with human quality of life (t = 5.162, *p* < 0.001), cognitive empathy (t = 3.109, *p* < 0.01), and affective empathy (t = 5.992, *p* < 0.001) in the dependent variable model. The mediation variable accounted for a substantial proportion of the variance in cognitive empathy (17.9%), affective empathy (38.2%), and human quality of life (7.5%). These findings support the significant dual-mediation effect (Table 4).

**Figure 2:**
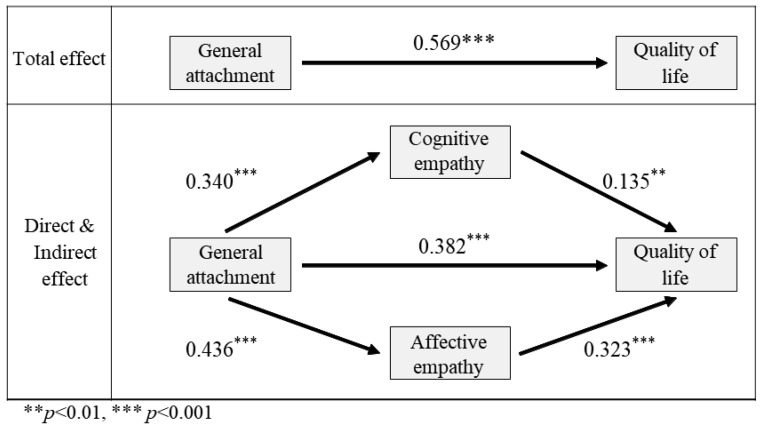
A schematic representation of the direct, indirect, and total dual-mediating effects of empathy on the relationship between general attachment and quality of life. Asterisks are used to indicate the significance level.

From Figure 2, it is evident that the total effect of general attachment on the quality of life of pet owners significantly decreased, as indicated by the statistical significance of dual mediation by two sub-factors of empathy. Therefore, it can be concluded that the nature of the parallel dual mediation effect is partial mediation.

### 3.5. The Parallel Dual-Mediation Effect of Empathy on the Relationship between Person Substitution and Human Quality of Life

A regression analysis was conducted to test H2 and examine the impact of empathy on the relationship between person substitution and human quality of life. F statistics were computed for each regression model, and all three models were deemed appropriate (Table 5). Model 1 revealed a significant influence of person substitution on cognitive empathy (t = 8.227, *p* < 0.001), while model 2 showed a significant effect on affective empathy (t = 6.391, *p* < 0.001). Both the dependent variable model and affective empathy had a positive effect on human quality of life (t = 6.818, *p* < 0.001; t = 6.935, *p* < 0.001, respectively), but cognitive empathy did not exhibit a significant effect (t = 1.200, *p* > 0.05). Bootstrap estimates confirmed the significance of these relationships and assessed the explanatory power of each model.

The regression analysis indicated that the mediation variables accounted for 20.6% and 13.5% of the variance in models 1 and 2, respectively, while the dependent variable model explained 42.2% of the variance.

To further explore the importance of each path in the regression model, which depicts how cognitive and affective empathy influence person substitution and human quality of life, bootstrapping was employed (Table 6). The path from person substitution to human quality of life had an effect size of 0.344. The mediation path mediated by cognitive empathy exhibited an effect size of 0.028, while the path mediated by affective empathy had an effect size of 0.126. However, the mediation path of cognitive empathy was found to be insignificant, with a range of −0.019 to 0.078 within a 95% confidence interval. In contrast, the mediation path of affective empathy was significant, with a range of 0.076 to 0.187, and zero was not included in the confidence interval. Thus, the study rejects Hypothesis 2, as the parallel dual-mediation effect was not significant.

**Figure 3:**
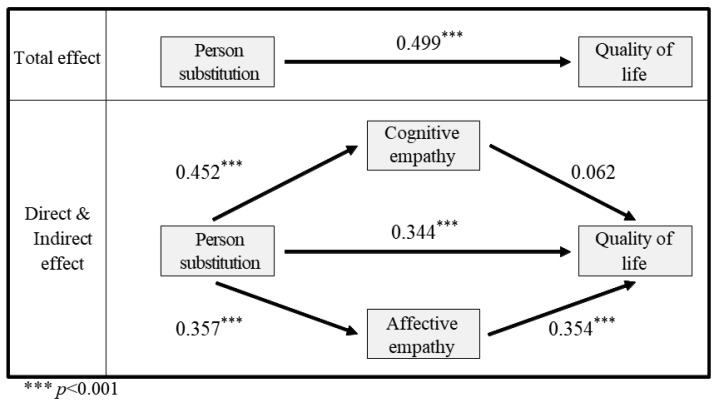
A schematic representation of the average dual-mediating effect of empathy on the relationship between Person substitution and quality of life. The asterisks indicate the significance level.

The results confirm the dual-mediation effect of cognitive and affective empathy on person substitution and human quality of life, as depicted in Figure 3. Including the two sub-factors of empathy as mediators decreased the total effect of person substitution on human quality of life. However, the path from cognitive empathy to human quality of life did not significantly influence the results. Therefore, the study concludes that there is no significant dual-mediation effect, as the path from cognitive empathy to human quality of life was found to be insignificant.

### 3.6. The Similar Dual-Mediating Effect of Empathy on the Relationship between General Attachment and Human Quality of Life

The study conducted a step-by-step regression analysis to test Hypothesis 3 and determine whether the two sub-factors of empathy had a similar dual-mediating effect on animal rights/welfare and human quality of life (Table 7). F-statistics were computed to assess the suitability of each regression model. All three regression models (model 1, model 2, and the dependent variable in model 3) were found to be appropriate, as indicated by the mediation variables. In model 1, animal rights/welfare exhibited a significant positive effect on cognitive empathy (t = 4.613, *p* < 0.001), while in model 2, it had a significant positive effect on affective empathy (t = 7.554, *p* < 0.001). The dependent variable model showed significant positive relationships between animal rights/welfare and human quality of life (t = 5.162, *p* < 0.001), as well as animal rights/welfare with cognitive (t = 3.109, *p* < 0.01) and affective empathy (t = 5.992, *p* < 0.001). Bootstrap estimation confirmed the significance of these relationships.

In this step-by-step regression analysis, the mediation variable accounted for 7.5%, 17.9%, and 38.2% of the variance in models 1, 2, and the dependent variable model, respectively.

The significance of each path in the regression model, regarding the dual-mediation effect of the two sub-factors of empathy in animal rights/welfare and human quality of life, was also verified (Table 8). The effect size of the path from animal rights/welfare to human quality of life was 0.239. The path from animal rights/welfare, mediated by cognitive empathy, had an effect size of 0.039, while the path mediated by affective empathy had an effect size of 0.125. The confidence intervals for both mediation paths did not include zero, indicating a significant parallel dual-mediation effect. Thus, the results support Hypothesis 3.

**Figure 4:**
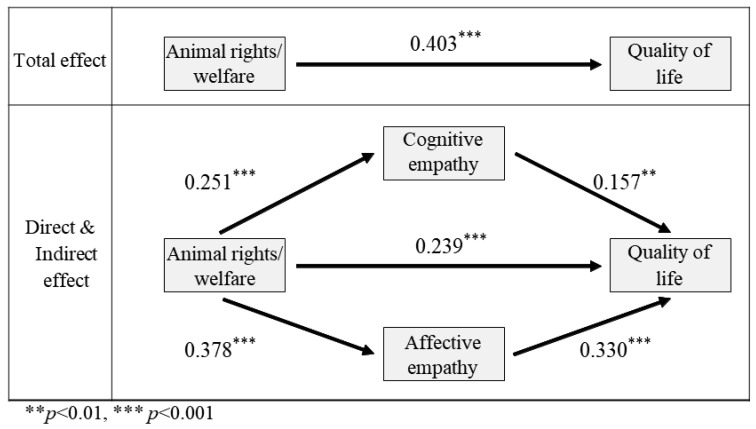
Schematic representation of the parallel dual-mediating effect of empathy on the relationship between animal rights/welfare and human quality of life. Asterisks indicate the significance level.

Based on the analysis above, the results confirm the similar dual-mediation effect of cognitive and affective empathy, the two sub-factors of empathy, in the relationship between animal rights/welfare and human quality of life, as depicted in Figure 4. It was observed that the total effect of animal rights/welfare on human quality of life significantly decreased when the two sub-factors of empathy were included as mediators. Therefore, the nature of the effect of parallel double mediation was identified as a partial mediation effect.

## 4. Discussion

The present study aimed to investigate the relationship between attachment to dogs, empathy, and human quality of life. The results provide valuable insights into the dual-mediation effect of empathy on attachment to dogs and human quality of life, as well as its influence on person substitution.

Our findings support the significant role of empathy in the relationship between human attachment and quality of life [2,4,10,27,49]. General attachment positively influences both cognitive and affective empathy, which significantly impact human quality of life. These results align with previous research highlighting the interconnectedness of empathy, attachment, and animal welfare [4,15,36,50,51].

Furthermore, our study demonstrates the partial mediation effect of empathy on the relationship between human attachment and quality of life [2,4,10]. The dual-mediation effect of cognitive and affective empathy partially mediates the relationship between general attachment and quality of life [2,49,52]. This underscores the importance of empathy in this relationship, acknowledging the presence of other contributing factors.

However, we did not find significant evidence of a parallel dual-mediation effect of empathy on the relationship between person substitution and quality of life. While both cognitive and affective empathy were found to have a significant effect on person substitution, only affective empathy exhibited a significant impact on human quality of life. This suggests a more complex role of empathy in the relationship between person substitution and quality of life than previously assumed [2,4,53]. 

The results imply that perceiving dogs as independent beings, rather than solely as substitutes for humans, may contribute to a broader understanding of empathy and its relationship with quality of life. This is supported by the significant impact of affective empathy on human quality of life, suggesting that recognizing the autonomy and individuality of dogs is important. However, it is important to note that further research and evidence are necessary to fully validate the claim that dogs should be viewed as independent entities.

We acknowledge the limitations of our study, which focused exclusively on adult dog owners in Korea, limiting the generalizability of the results. To gain a comprehensive understanding of human–animal interactions, future research should include a diverse range of pet owners from various geographical and cultural backgrounds, encompassing different pet species and considering cultural preferences for specific dog breeds.

Conducting more comprehensive and inclusive studies will further advance our understanding of the intricate relationships between attachment, empathy, and animal welfare, as well as their impact on human quality of life. This knowledge will inform interventions and policies aimed at promoting the well-being of both humans and animals in broader contexts.

## 5. Conclusions

To summarize, this study sheds light on the significant role of empathy in the attachment between humans and animals, and its profound impact on human quality of life. Firstly, it underscores the importance of empathy in human–animal attachment and its considerable influence on human well-being. Secondly, both cognitive and affective empathy play crucial roles in mediating the relationship between human attachment and quality of life. Thirdly, our findings indicate that general attachment positively affects both cognitive empathy and affective empathy, which in turn significantly contribute to human quality of life.

Furthermore, empathy acts as a partial mediator in the relationship between human attachment and quality of life, suggesting a complex interplay among various factors. It is crucial to acknowledge the involvement of additional contributing factors and consider the limitations of this study, such as its exclusive focus on adult dog owners in Korea.

Future studies should aim for greater diversity by including pet owners from diverse backgrounds and cultural contexts, while also considering a variety of pet species and cultural preferences. Conducting more comprehensive studies will deepen our understanding of the intricate dynamics between attachment, empathy, and their impact on human quality of life.

## Figures and Tables

**Table 1:** Empathy and attachment sub-factors for normality testing.

Factors	M	SD	Skewness	Kurtosis
**Pet Attachment**	4.19	0.590	−0.870	0.472
General attachment	4.35	0.590	−0.917	0.363
Person substitution	4.01	0.680	−0.692	0.396
Animal rights/welfare	4.21	0.750	−1.143	1.038
**Empathy**	3.52	0.570	0.490	0.052
Cognitive empathy	3.55	0.680	−0.026	−0.027
Affective empathy	3.50	0.660	0.107	−0.243
Quality of life *	3.66	0.650	−0.013	0.114

M, mean, SD, standard deviation. * World Health Organization Quality of Life-BREF (WHOQOL-BREF) [44].

**Table 2:** Correlation analysis of attachment and empathy sub-factors and human quality of life.

Factors	a	b	c	d	e	f
a. General attachment	1					
b. Person substitution	0.660 **	1				
c. Animal rights/welfare	0.730 **	0.632 **	1			
d. Cognitive empathy	0.293 **	0.454 **	0.275 **	1		
e. Affective empathy	0.385 **	0.368 **	0.424 **	0.421 **	1	
f. Quality of life	0.525 **	0.537 **	0.472 **	0.390 **	0.534 **	1

** *p* < 0.01; different lowercase letters in the first row indicate a, general attachment; b, person substitution; c, animal rights/welfare; d, cognitive empathy; e, affective empathy; f, quality of life.

**Table 3:** Verification of the parallel dual-mediation effects of empathy on the relationship between general attachment and human quality of life.

Model	Sub-Factor	B	SE	t	*p*	LLCI †	ULCI ‡
Parameter model 1 (dependent variable: cognitive empathy)	(Constant)	2.068	0.301	6.862	0.000 ***	1.475	2.662
	General attachment	0.340	0.069	4.949	0.000 ***	0.205	0.475
Parameter model 2 (dependent variable: affective empathy)	(Constant)	1.603	0.284	5.645	0.000 ***	1.044	2.162
	General attachment	0.436	0.065	6.732	0.000 ***	0.308	0.563
Dependent variable model (dependent variable: quality of life)	(Constant)	0.396	0.249	1.593	0.112	−0.094	0.885
	General attachment	0.382	0.056	6.818	0.000 ***	0.272	0.493
	Cognitive empathy	0.135	0.049	2.753	0.006 **	0.039	0.232
	Affective empathy	0.323	0.052	6.181	0.000 ***	0.220	0.425
F = 24.494 (*p* < 0.001), R^2^ = 0.086/F = 45.319 (*p* < 0.001), R^2^ = 0.148/F = 63.154 (*p* < 0.001), R^2^ = 0.422							

** *p* < 0.01, *** *p* < 0.001; B, unstandardized coefficient; SE, standard error; T, test statistics † LLCI: the lower bound within the 95% confidence interval of the bootstrap indirect effect; ‡ ULCI: the upper bound within the 95% confidence interval of the bootstrap indirect effect.

**Table 4:** Verification of the significance of empathy’s indirect effects on the relationship between general attachment and human quality of life.

Classification	Effect	SE	95% Confidence Interval	
			LLCI †	ULCI ‡
General attachment → Quality of life	0.382	0.056	0.272	0.493
General attachment → Cognitive empathy → Quality of life	0.046	0.020	0.011	0.090
General attachment → Affective empathy → Quality of life	0.141	0.033	0.082	0.210

† LLCI: the lower bound within the 95% confidence interval of the boot indirect effect; ‡ ULCI: the upper bound within the 95% confidence interval of the boot indirect effect; SE, standard error.

**Table 5:** Verification results of the parallel dual mediating effect of empathy ability on the relationship between Person substitution and human quality of life.

Model	Subfactor	B	SE	t	*p*	LLCI †	ULCI ‡
Parameter model 1 (dependent variable: cognitive empathy)	(Constant)	1.736	0.223	7.773	0.000 ***	1.296	2.175
	Person substitution	0.452	0.055	8.227	0.000 ***	0.344	0.560
Parameter model 2 (dependent variable: affective empathy)	(Constant)	2.065	0.227	9.080	0.000 ***	1.617	2.513
	Person substitution	0.357	0.056	6.391	0.000 ***	0.247	0.467
Dependent variable model (dependent variable: quality of life)	(Constant)	0.830	0.213	3.905	0.000 ***	0.412	1.249
	Person substitution	0.344	0.050	6.818	0.000 ***	0.245	0.443
	Cognitive empathy	0.062	0.052	1.200	0.231	−0.040	0.165
	Affective empathy	0.354	0.051	6.935	0.000 ***	0.253	0.454
F = 67.682 (*p* < 0.001), R^2^ = 0.206/F = 40.850 (*p* < 0.001), R^2^ = 0.135/F = 63.153 (*p* < 0.001), R^2^ = 0.422							

*** *p* < 0.001; † LLCI: the lower bound within the 95% confidence interval of the bootstrap indirect effect; ‡ ULCI: the upper bound within the 95% confidence interval of the bootstrap indirect effect; B, unstandardized coefficient; SE, standard error; T, test statistics.

**Table 6:** The significant verification of indirect empathy effects on the relationship between person substitution and human quality of life.

Classification	Effect	SE	95% Confidence Interval	
			LLCI †	ULCI ‡
Person substitution → Quality of life	0.344	0.050	0.245	0.443
Person substitution → Cognitive empathy → Quality of life	0.028	0.024	−0.019	0.078
Person substitution → Affective empathy → Quality of life	0.126	0.028	0.076	0.187

† LLCI: the lower bound within the 95% confidence interval of the boot indirect effect. ‡ ULCI: the upper bound within the 95% confidence interval of the boot indirect effect; SE, standard error.

**Table 7:** Verification of the parallel dual-mediation effects of empathy on the relationship between animal rights/welfare and human quality of life.

Model	Sub-Factor	B	SE	t	*p*	LLCI †	ULCI ‡
Parameter model 1 (dependent variable: cognitive empathy)	(Constant)	2.489	0.233	10.693	0.000 ***	20.031	20.947
	Animal rights/welfare	0.251	0.054	4.613	0.000 ***	0.144	0.358
Parameter model 2 (dependent variable: affective empathy)	(Constant)	1.905	0.214	8.903	0.000 ***	1.484	2.327
	Animal rights/welfare	0.378	0.050	7.554	0.000 ***	0.279	0.476
Dependent variable model (dependent variable: quality of life)	(Constant)	0.950	0.222	4.275	0.000 ***	0.512	1.388
	Animal rights/welfare	0.239	0.046	5.162	0.000 ***	0.148	0.330
	Cognitive empathy	0.157	0.051	3.109	0.002 **	0.058	0.257
	Affective empathy	0.330	0.055	5.992	0.000 ***	0.221	0.438
F = 21.281 (*p* < 0.001), R^2^ = 0.075/F = 57.057 (*p* < 0.001), R^2^ = 0.179/F = 53.444 (*p* < 0.001), R^2^ = 0.382							

** *p* < 0.01, *** *p* < 0.001; † LLCI: the lower bound within the 95% confidence interval of the bootstrap indirect effect; ‡ ULCI: the upper bound within the 95% confidence interval of the bootstrap indirect effect; B, unstandardized coefficient; SE, standard error; T, test statistics.

**Table 8:** Significance verification of indirect effects of sympathy on the relationship between animal rights/welfare and human quality of life.

Classification	Effect	SE	95% Confidence Interval	
			LLCI †	ULCI ‡
Animal rights/welfare → Quality of life	0.239	0.046	0.148	0.330
Animal rights/welfare → Cognitive empathy → Quality of life	0.039	0.017	0.012	0.077
Animal rights/welfare → Affective empathy → Quality of life	0.125	0.030	0.072	0.188

† LLCI: the lower bound within the 95% confidence interval of the boot indirect effect. ‡ ULCI: the upper bound within the 95% confidence interval of the boot indirect effect; SE, standard error.

## Data Availability

The data presented in this study are available on request from the author for scientific purposes.

## Supplementary Materials

The following supporting information can be downloaded at: https://www.mdpi.com/article/10.3390/ani13132220/s1. Table S1. Study participant sociodemographic data (n = 263); Table S2. Results of Lexington attachment to pets scale factor and reliability analyses. Table S3. Results of interpersonal reactivity index factor and reliability analysis.
