# Supplementary material for: Exploring the Role of Empathy as a Dual Mediator in the Relationship between Human–Pet Attachment and Quality of Life: A Survey Study among Adult Dog Owners
Source: Animals (Basel). 2023 Jul 6;13(13):2220. doi: 10.3390/ani13132220 (PMC10339944; doi:10.3390/ani13132220)
Supplement: Supplementary file 1 [file animals-13-02220-s001.zip › animals-2448053-supplementary.pdf]

animals

MDPI

Table S1. Study participant sociodemographic data (n = 263).

Variables	Category	Frequency (N)	Percentage (%)
Gender	Male	97	36.9
	Female	166	63.1
Age group	20s	69	26.2
	30s	154	58.6
	40s	29	11
	50s	11	4.2
Education level	High school or lower	25	9.5
	College	51	19.4
	Undergraduate degree	177	67.3
	Master's degree or higher	10	3.8
Occupation	Student	13	4.9
	Office worker	186	70.7
	Self-employed	26	9.9
	Freelancer	18	6.8
	Homemaker	11	4.2
	Unemployed	9	3.4
Marital status	Married	65	24.7
	Unmarried	198	75.3
Monthly income	Under $200	39	14.8
	$1001–$1500	135	51.3
	$1501–$2000	62	23.6
	Over $2001	27	10.3
Parenting experience	First-time parents	213	81
	Second over	50	19
Breed of dog	Maltese	50	19
	Pomeranian	50	19
	Poodle	53	20.2
	Mixed breed	53	20.2
	Shih Tzu	26	9.9
	Chihuahua	13	4.9
	Bichon Prize	11	4.2
	Other	7	2.7
Number of pet dogs owned	One dog	250	95.1
	Two or more dogs	13	4.9
Dog age (years)	Under 3 years old	125	47.5
	4–6 years old	85	32.3
	7–9 years old	25	9.5
	Over 10 years old	28	10.6
Period of upbringing (dog age)	Under 3 years old	142	54
	4–6 years old	67	25.5
	7–9 years old	20	7.6
	Over 10 years old	34	12.9
	Total	263	100

Table S2. Results of Lexington attachment to pets scale factor and reliability analyses.

Variables		Factors 1	2	3	Eigenvalue (Descriptive Variance)	Cronbach's α
General attachment	attachment2	0.820			5.640 (24.521)	0.921
	attachment4	0.774				
	attachment3	0.728				
	attachment6	0.675				
	attachment8	0.647				
	attachment10	0.639				
	attachment5	0.608				
	attachment7	0.586				
	attachment11	0.584				
	attachment1	0.583				
	attachment9	0.557				
Person substitution	attachment17		0.726		4.190 (18.218)	0.841
	attachment13		0.720			
	attachment18		0.705			
	attachment16		0.627			
	attachment15		0.620			
	attachment14		0.573			
	attachment12		0.525			
Animal rights/welfare	attachment22			0.824	3.739 (16.257)	0.860
	attachment21			0.676		
	attachment20			0.639		
	attachment19			0.637		
	attachment23			0.619		

Table S3. Results of interpersonal reactivity index factor and reliability analysis.

Variables		Factors		Eigenvalue (Descriptive Variance)	Cronbach's α
		1	2		
Cognitive empathy	empathy2	0.822		6.318 (22.564)	0.899
	empathy7	0.813			
	empathy4	0.755			
	empathy5	0.75			
	empathy13	0.721			
	empathy1	0.698			
	empathy11	0.604			
	empathy9	0.574			
	empathy8	0.552			
	empathy14	0.551			
	empathy3	0.549			
	empathy10	0.545			
	empathy6	0.513			
	empathy12	0.508			
Affective empathy	empathy19		0.82	5.942 (21.222)	0.881
	empathy20		0.738		
	empathy16		0.672		
	empathy18		0.666		
	empathy21		0.607		
	empathy22		0.603		
	empathy28		0.601		
	empathy17		0.576		
	empathy26		0.566		
	empathy15		0.565		
	empathy24		0.555		
	empathy27		0.549		
	empathy25		0.53		
	empathy23		0.522		
